# Molecular Characterization and Genomic Function of Grapevine Geminivirus A

**DOI:** 10.3389/fmicb.2020.555194

**Published:** 2020-09-02

**Authors:** Suwei Sun, Ya Hu, Guangzhuang Jiang, Yimin Tian, Ming Ding, Cui Yu, Xueping Zhou, Yajuan Qian

**Affiliations:** ^1^Institute of Biotechnology, Zhejiang University, Hangzhou, China; ^2^Technical Center for Animal, Plant and Food Inspection and Quarantine, Shanghai Customs District, Shanghai, China; ^3^Institute of Biotechnology and Germplasm Resources, Yunnan Academy of Agricultural Science, Kunming, China; ^4^Institute of Plant Protection, Chinese Academy of Agricultural Sciences, Beijing, China

**Keywords:** grapevine geminivirus A, defective subviral molecule, pathogenicity, genetic variability, genetic evolution

## Abstract

A new grapevine geminivirus A (GGVA) isolate (named as GGVA-17YM1) and its associated defective genome (GGVA-D) were identified from a grapevine sample collected in Yuanmou, Yunnan Province, using sRNA high throughput sequencing and traditional Sanger sequencing. To explore the pathogenicity of GGVA and GGVA-D, infectious clones of GGVA-17YM1 and GGVA-D-17YM1 were constructed. Infection assays indicated that *Nicotiana benthamiana* plants inoculated with GGVA alone or a combination of GGVA and GGVA-D exhibited upward curled apical leaves and dwarfism. Southern blotting and quantitative real-time polymerase chain reaction analysis revealed that GGVA-D increased the accumulation level of GGVA DNA. Transient expression using a PVX-derived recombinant vector indicated that C2 and C4 encoded by GGVA are involved in symptom induction in *N. benthamiana*. Furthermore, the V2 protein inhibited local RNA silencing in co-infiltration assays in *GFP* transgenic *N. benthamiana* plants. Subsequently, full-length genome sequencing resulted in the identification of 11 different isolates of GGVA and 9 associated defective DNA molecules. Phylogenetic analysis based on whole genome sequences showed that all GGVA isolates, including our sequences, clustered into two distinct branches with no geographical grouping. Analyses of molecular variation indicated single nucleotide polymorphisms (SNPs) with more transitions (55.97%) than transversions (44.03%). Furthermore, the main variants for ORF C1, C3, or V1 were synonymous mutations, and non-synonymous mutations for ORF C2, C4, and V2. Genetic selection analysis indicated that negative selection acted on four ORFs (V1, C1, C2, and C3), while V2 and C4 were under positive selection. Our results contribute to the characterization of the genetic diversity of GGVA and provide insights into its pathogenicity.

## Introduction

Grape (*Vitis* spp.) is an important fruit crop worldwide. Grape berries are consumed as fresh fruit or used for juice, wine, jam, and other byproducts. Years of asexual reproduction have resulted in the presence of many pathogens in grapevines, some of which can cause serious yield losses and reduction in quality (Mostert et al., 2006). More than 65 viruses have been found in grapevines (Martelli, 2014) and multiple viral infections are frequently present in symptomatic or asymptomatic vines in vineyards. Most grapevine-infecting viruses have ribonucleic acid (RNA) genomes (Al Rwahnih et al., 2017). However, several DNA viruses have also been found in grapevines, such as grapevine vein clearing virus (GVCV) (Zhang et al., 2011), grapevine Roditis leaf discoloration associated virus (GRLDaV) (Maliogka et al., 2015), grapevine red blotch virus (GRBV) (Al Rwahnih et al., 2013, 2015), and grapevine geminivirus A (GGVA) (Al Rwahnih et al., 2017). GRBV is the type number of the genus *Grablovirus* in the family *Geminiviridae*, while GGVA is a tentative member of this family (Krenz et al., 2014; Al Rwahnih et al., 2017; Varsani et al., 2017; Cieniewicz et al., 2018).

Geminiviruses have a circular, single-stranded DNA genome (ssDNA) packed in twin incomplete icosahedrons (Bock et al., 1974; Harrison et al., 1977). Bipartite geminiviruses have two approximately 2.6 Kb-sized ssDNA components referred to as DNA-A and DNA-B (Haber et al., 1983; Hamilton et al., 1983; Stanley and Gay, 1983) and monopartite geminiviruses have only one component resembling DNA-A (Ber et al., 1990; Navot et al., 1991). Geminiviruses are currently classified into nine different genera (*Grablovirus*, *Capulavirus*, *Begomovirus*, *Mastrevirus*, *Topocuvirus*, *Curtovirus*, *Becurtovirus*, *Eragrovirus*, and *Turncurtovirus*) based on host range, type of insect vector and phylogenetic relationships (Zerbini et al., 2017). Evidence indicates that most monopartite begomoviruses are associated with satellite DNA (Zhou, 2013) or subgenomic defective DNA comprising half the size of the genomic components (Al Rwahnih et al., 2017). Previous reports showed that these subgenomic components might be involved in the regulation of virus replication or have a causative role in viral pathogenicity (Behjatnia et al., 2007).

GGVA and its defective DNA (GGVA-D) were first found in 2017 through high throughput sequencing (Al Rwahnih et al., 2017). The complete genome of GGVA ranges from 2903 to 2907 nucleotides in length, including two open reading frames (ORFs) (V1, coat protein; V2, putative movement protein) on the viral-sense strand and four ORFs (C1, replication associated protein; C2, transcriptional activator protein, C3, replication enhancer, and C4, host activated protein) on the complementary strand. The GGVA-D sequence corresponds to about 54% of GGVA in length, including the encoded sequences of *V1*, *V2*, and the N-terminal partial sequence of *C1*. The genomes of GGVA and GGVA-D contain a conserved stem-loop sequence motif “TAATATTAC” and an intergenic region (IR) (Fan et al., 2017; Jo et al., 2018). To the best of our knowledge, a GGVA-D related sequence characterized from grapevines has only been associated with GGVA. Additionally, very little is known about the molecular biology of GGVA and GGVA-D.

In the present study, we first identified GGVA isolates in two previously unreported locations, Yunnan and Shanghai, China and confirmed that GGVA-D was usually associated with GGVA in these isolates. Infectious DNA clones of GGVA and GGVA-D were constructed and inoculated into *Nicotiana benthamiana* via *Agrobacterium*-mediated delivery to show that GGVA causes disease symptoms and that GGVA-D increases the accumulation level of GGVA. Additionally, we investigated the role of certain predicted proteins encoded by GGVA. Our results not only provide valuable molecular characterization of a GGVA population, but also contribute to our understanding of the pathogenicity of GGVA and GGVA-D.

## Materials and Methods

### Virus Resources

In September, 2017, nine samples from peach, apricot, citrus, walnut, grapevine, and mango showing yellowing, curly mottled, crinkled or mosaic leaves were collected in Lüliang, Shilin, and Yuanmou, Yunnan Province, China (Supplementary Table S1). In the spring and summer of 2018 and 2019, 18 grapevine leaf samples showing virus-like chlorotic ringspot, crinkled, and yellowing symptoms, or asymptomatic symptoms were collected from seven vineyards in Pudong, Shanghai, and Yuanmou, Yunnan (Supplementary Table S2).

### Small RNA Deep Sequencing

A 0.2 g sample of mixed leaf tissues selected from different fruit crops was used for small RNA sequencing using a Hiseq2500 sequencer (Illumina, San Diego, CA, United States) (Metzker, 2010). The raw data were filtered and cleaned using an in-house Perl script. The 18–28 nt reads consisting of trimmed sRNA sequences were collected for subsequent analysis. The Velvet program (EMBL-EBI, Cambridge, United Kingdom, Public Git URL: git clone git://github.com/dzerbino/velvet.git) was used for genome assembly and a parameter of 17 nucleotides was set as the minimal overlapping length (k-mer) to join two sRNAs into a contig (Zerbino and Birney, 2008). Contig sequences were subjected to BLASTn (nucleotide BLAST) alignment in the NCBI (the National Center for Biotechnology Information) database.

### Construction of Infectious Clones

A recombinant plasmid containing 1.8 copies of the full-length fragment of the viral genome (Grimsley et al., 1987), including the two IR regions, was constructed. First, a 2904 bp full-length and a 2500 bp partial GGVA fragment were amplified from a 17YM1 grapevine sample using high-fidelity PCR (Vazyme, Nanjing, China) with primers GGVA-1-(2885)-F/GGVA-1-(2884)-R or GGVA-2-(2885)-F/GGVA-2 -(2480)-R (Primer sequences are listed in Supplementary Table S3) and ligated into the pclone007 vector (Tsingke, Beijing, China) to produce constructs GGVA-1-pclone007 and GGVA-2-pclone007. After sequencing, the fragments of GGVA-1 and GGVA-2 were obtained by digestion using *Sal*I/*Bcu*I or *Sal*I/*Kpn*I (Thermo, Waltham, MA, United States), respectively. The resulting fragment of GGVA-2 was cloned into the binary vector pBinplus digested by *Sal*I and Kpnl to produce GGVA0.8-pBinplus. Subsequently, the fragment of GGVA-1 was inserted into GGVA0.8-pBinplus digested by *Sal*I and *Bcu*I, resulting in GGVA1.8-pBinplus. The clone of GGVA-D-1.8-pBinplus was constructed using the same strategy.

### Construction of a Transient Expression Vector

Using recombinant plasmid GGVA1.8-pBinplus as a template, six target fragments (V1, V2, C1, C2, C3, and C4) were amplified using high-fidelity PCR with specific primer pairs (Supplementary Table S3). The resulting fragments were digested with *Asc*I and *Sal*I (Thermo) and inserted into the PGR106 vector, or were cloned into the binary vector pCHF3 by homologous recombination, separately.

### Agroinoculation of *N. benthamiana* Plants

The recombinant plasmids GGVA1.8-pBinplus and GGVA-D-1.8-pBinplus were transformed into *Agrobacterium tumefaciens* EHA105 using electroporation. The PGR106-based expression vectors were individually transformed into *A. tumefaciens* GV3101, while the PCHF3-based vectors were individually transformed into *A. tumefaciens* C58C1. *A. tumefaciens* cultures harboring the different constructs were grown at 28°C overnight in YEP medium until the OD_600_ reached approximately 0.8. Before infiltration, individual agrobacterium cultures were resuspended in induction buffer (10 mM MES, pH 5.7, 10 mM MgCl_2_, 200 mM acetosyringone) to a final OD_600_ = 1.0. In addition, for the co-inoculation of different infectious components, the following groups were used: (1) GGVA + pBinplus; (2) GGVA-D + pBinplus; (3) GGVA + GGVA-D; (4) pBinplus alone used as the control group. Equal volumes of the separate agrobacterial cultures (OD_600_ = 1.0) were mixed before inoculation. Four to six expanded leaves of *N. benthamiana* plants were agroinoculated with agrobacterial cultures using needleless 1 mL syringes. Inoculated plants were cultured in a growth chamber at 25°C under a 16 h–8 h light/dark cycle.

### RCA and Enzyme Digestion

The DNA samples were enriched using rolling circle amplification (RCA) with an RCA TempliPhi 100 Amplification Kit (GE Healthcare, Little Chalfont, Buckinghamshire, United Kingdom) according to the manufacturer’s instructions. After a long amplification for 18 h at 30°C, the product was heated for 10 min to deactivate the enzyme and then subjected to restriction enzyme digestion using *Bam*HI (Thermo) for 1 h at 37 °C. The products were analyzed by electrophoresis to verify the existence of GGVA or its defective genome GGVA-D.

### Quantitative Real-Time PCR (qPCR) Analysis, Reverse Transcription-PCR (RT-PCR)

The DNA concentration was quantified and adjusted at 500 ng/μL. The plant 25S rDNA gene amplified using specific primers q25S-F and q25S-R was used as the reference gene. The specific primer pair for GGVA was qPCR-GGVA-1560-F/qPCR-GGVA-1780-R, which did not amplify a fragment of the defective GGVA-D (Supplementary Table S3). The LightCycler^®^ 480 SYBR Green I Master mix (Roche, Basel, Switzerland) was applied for qPCR analysis according to the manufacturer’s instructions. The qPCR program was 95 °C for 5 min, followed by 45 cycles of 95°C for 10 s, 60°C for 10 s, and 72°C for 10 s. The relative amounts of the target genes were computed using the LightCycler^®^ 480 Gene Scanning Software. qRT-PCR analysis was used to determine GFP mRNA levels, and the glyceraldehyde-3-phosphate dehydrogenase (GADPH) gene amplified using specific primers qPCR-GADPH-F and qPCR-GADPH-R was used as the reference gene. The specific primer pair for GFP was qPCR-GFP-F/qPCR-GFP-R (Supplementary Table S3).

Total RNA was extracted from leaf tissues using the TRIzol reagent (Invitrogen, Carlsbad, CA, United States) according to the manufacturer’s instructions. The extracted RNA was reverse transcribed into cDNA using ReverTra Ace qPCR RT Master Mix with gDNA Remover (TOYOBO, Shanghai, China) following the manufacturer’s instructions. The cDNA products were amplified using high-fidelity PCR (Vazyme, Nanjing, China) and specific primers (Supplementary Table S3).

### Protein Extraction and Western Blotting

Total protein was extracted from leaf tissues using SDS-urea buffer and then separated using 15% SDS-PAGE. After transferring the proteins to a solid phase nitrocellulose membrane (GE Healthcare, Little Chalfont, Buckinghamshire, United Kingdom), an antibody-specific assay was carried out with rabbit anti-PVX CP polyclonal antibodies (prepared in our laboratory) or with rabbit anti-GFP monoclonal antibodies (Epitomics, Burlingame, CA, United States) followed by a goat anti-rabbit IgG conjugated with alkaline phosphatase (Sigma-Aldrich, St. Louis, MI, United States). The signals from the immunoreactive proteins were visualized using a nitro blue tetrazolium (NBT)-5-Bromo-4-chloro-3-indolyl phosphate (BCIP) solution (Promega, Madison, WI, United States).

### DNA Extraction and Southern Blotting

DNA was extracted from apical *N. benthamiana* leaves using the cetyltrimethylammonium bromide (CATB) method, separated by agarose gel electrophoreses, transferred to Hybond-N membranes (GE Healthcare) and cross-linked. Following alkali denaturation and neutralization, the immobilized DNA was hybridized with a Digoxin-labeled 856 bp probe specific for the GGVA genome but not for the GGVA-D genome. The detected signals were visualized using a Detection Starter Kit II (Roche, Basel, Switzerland) according to the manufacturer’s instructions.

### Phylogenetic Analysis

Multiple nucleotide sequence alignments were performed using the MUSCLE algorithm available in MEGA version X (Kumar et al., 2018). The maximum -likelihood (ML) method was used to construct the phylogenetic tree (Saitou and Nei, 1987). The tree was evaluated using a bootstrap test with 1000 replicates. The sequences derived from tomato leaf curl Madagascar virus (ToLCMGV) or tomato yellow leaf curl China virus (TYLCCNV) served as outgroups. The sequences that were selected and obtained from the NCBI database for the phylogenetic analysis are listed in Supplementary Table S4.

### Genomic Sequence Analysis

The extent of GGVA variation among sequences from different sources was analyzed using DNA Sequence Polymorphism analysis software version 6 (DnaSP 6) (Librado and Rozas, 2009), setting a window length of 100 and a step size of 25. The mutation rate of a single nucleotide was evaluated based on the nucleotide diversity parameter (π). The base transitions/transversion ratios for the GGVA genome were estimated using the maximum composite likelihood method in MEGA 5 (Tamura et al., 2011).

The occurrence of selection acting on different GGVA ORFs was estimated at the Datamonkey website (Weaver et al., 2018)^1^ using three methods. The dn/ds is the average ratio between non-synonymous and synonymous substitutions for each pair of comparisons. FEL (Fixed Effects Likelihood) uses a maximum-likelihood (ML) approach; SLAC (Single-Likelihood Ancestor Counting) uses a combination of ML and counting approaches; and FUBAR (Fast, Unconstrained Bayesian AppRoximation) uses a Bayesian approach.

## Results

### GGVA Was Identified Through Small RNA Deep Sequencing

**FIGURE 1:**
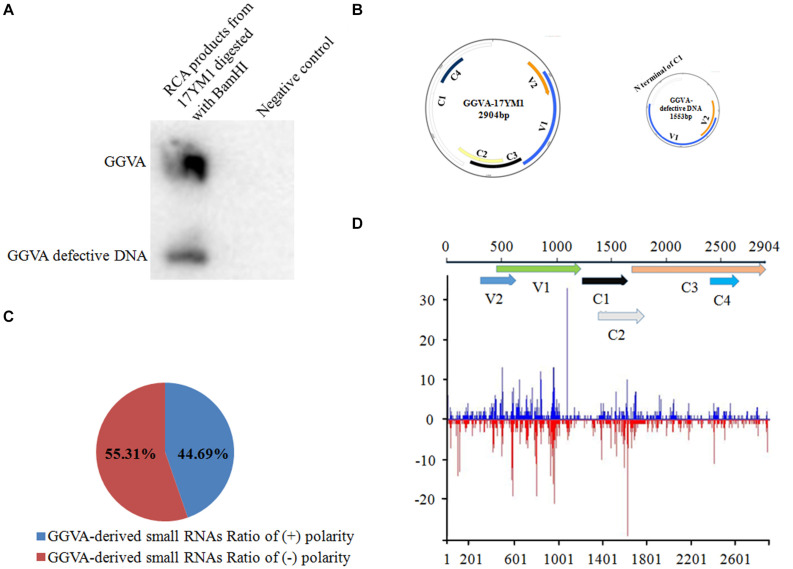
Identification of grapevine geminivirus A (GGVA) and characterization of viral derived siRNAs from GGVA. **(A)** Southern blotting to verify the presence of GGVA and its defective DNA in the field grapevine sample named 17YM1. **(B)** Schematic representation of GGVA isolate 17YM1 and its defective DNA genome structures predicted by SnapGene. V1, coat protein; V2, pre-coat protein; C1, replication-associated protein; C2, transcriptional activator protein; C3, replication enhancer protein; C4, host activator protein; IR, intergenic region sequences which was 569 bp, a stem-loop structure contained a conserved 9-base nucleotide sequence “TAATATTAC” similar to other geminiviruses. **(C)** Polarity distribution of the GGVA-derived small RNAs derived from positive-sense strands (+) or negative-sense strands (–) using the Perl script. **(D)** Distribution frequency of viral small RNA along the GGVA-17YM1 genome.

Nine samples from different fruit crops showing virus-like symptoms in the field were mixed and analyzed using unbiased high throughput Illumina sequencing. A total of 4935 contigs were assembled from the sRNA library using the software package Velvet. Subsequently, these contigs were submitted for BLASTn analysis. The comparisons demonstrated that one of the 4935 contigs showed high similarity with GGVA (93–95% nucleotide sequence identity). To further confirm the presence of GGVA, primers were designed based on the identified contig and a resulting 372 bp fragment was obtained by PCR only from the grapevine sample named 17YM1. None of the other fruit crop samples yielded a PCR product. Additionally, RCA using the crude extract of total DNA from sample 17YM1 as a template, followed by restriction fragment length polymorphism analyses, identified two specific fragments of 1.5 kb or 3.0 kb in length. Accordingly, Southern blotting analysis with a GGVA-specific probe validated the RCA products digested by *Bam*HI and identified the presence of GGVA and its defective component GGVA-D in sample 17YM1 (Figure 1A). Further back-to-back primers based on the complete sequences of GGVA and its defective DNA downloaded from the NCBI database were designed to amplify the full-length genomic sequences of GGVA and GGVA-D from sample 17YM1, yielding fragments of approximately 3.0 or 1.5 kb. Following Sanger sequencing, a BLASTn search in the NCBI database identified these two sequences as new isolates of GGVA and its associated defective DNA, and the names of GGVA-17YM1 (GenBank accession number MT344703) and GGVA-D-17YM1 (GenBank accession number MT344715) were adopted. According to online prediction^2^, a stem-loop structure containing a conserved 9-base nucleotide sequence “TAATATTAC,” which is the typical feature found in geminiviruses, is present in the genome of GGVA-17YM1 and GGVA-D-17YM1. SnapGene analysis predicted a 569 bp-sized intergenic region (IR) and six ORFs in the GGVA-17YM1 genomic sequence, including V1 (771 bp), V2 (312 bp), C1 (1212 bp), C2 (420 bp), C3 (429 bp), and C4 (258 bp). Moreover, three ORFs were predicted in the GGVA-D-17YM1 genomic sequence, showing high homology to the V1, V2, and the N-terminus of C1 of GGVA-17YM1, respectively (Figure 1B).

### Characterization of Viral Derived siRNAs From GGVA

The profile of viral small interfering RNAs (vsiRNAs) derived from a specific viral genome provides strong evidence for the presence of this virus. Using Bowtie tools with zero mismatches allowed, a total of 2835 vsiRNAs were found to be perfectly mapped along the genome of GGVA isolate 17YM1. Furthermore, analysis of the polarity distribution of these vsiRNAs indicated a moderate excess (55.31%) toward the negative strand, suggesting a slightly asymmetric distribution on the genome (Figure 1C). A genome-wide view of vsiRNAs revealed that the GGVA vsiRNAs covered almost the entire genome of GGVA, with the main hotspot profile along the coding regions of V1 and V2 and the coding regions of C2 and C3 (Figure 1D).

### Infectivity of GGVA and GGVA-D

**FIGURE 2:**
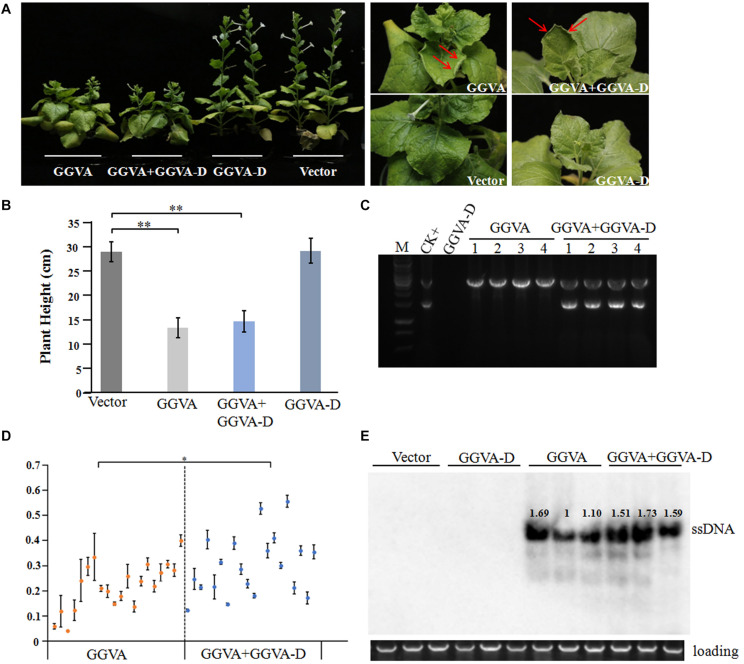
Systemic infection of GGVA and GGVA-D in *Nicotiana benthamiana*. **(A)** Symptoms induced by GGVA and GGVA-D alone or in combined infection in *N. benthamiana* plants. The red arrow points to a curled leaf. **(B)** Height of *N. benthamiana* infected by GGVA and GGVA-D alone or in combination. Significant differences assessed using a *T*-test (*P* < 0.01) are shown by ^∗∗^. **(C)** The electrophoresis diagram of digested RCA products with *Bam*HI that derived from GGVA and GGVA-D alone or combined infection in *N. benthamiana* plants. The result of the field sample 17YM1 was used as the positive control. **(D)** Analysis of the accumulation level of GGVA in *N. benthamiana* plants infected by GGVA alone or by GGVA/GGVA-D together at 30 dpi using qPCR. Each scatter point in the figure represents a biological replicate, and the error line was drawn for the three technical replicates of each biological replicate. Significant differences assessed using a *T*-test (0.01 < *P* < 0.05)are indicated by ^∗^. **(E)** Accumulation of GGVA in inoculated plants as detected using Southern blotting hybridization with a probe specific to the GGVA genome. Three biological replicates for per inoculation column were set.

Following agroinoculation with the GGVA infectious clone alone or combined with the GGVA-D clone in the experimental host *N. benthamiana*, it was clearly observed that all the inoculated plants developed dwarfism, upward curling of leaves and delayed flowering at 30 days post-inoculation (dpi) (Figures 2A,B). Noticeably, co-agroinoculation with GGVA and GGVA-D did not aggravate the symptoms in *N. benthamiana* plants compared with GGVA single infection. Additionally, no disease symptom was observed in *N. benthamiana* inoculated with the GGVA-D clone compared with agroinoculation with an empty binary vector.

To further verify the presence of viral DNA in all inoculated plants, total DNA from apical leaves was extracted and used as a template for RCA combined with *Bam*HI digestion. A specific fragment of about 3 kb was obtained from DNA samples isolated from plants inoculated with GGVA while two fragments of 1.5 kb and 3 kb were obtained from the samples co-inoculated with GGVA and GGVA-D. However, in the case of single agroinoculation with GGVA-D, no products were detected (Figure 2C). Further assays indicated that GGVA infects *N. benthamiana* at a rate of 100%, regardless of whether these plants were co-inoculated with GGVA/GGVA-D or inoculated with the GGVA alone, as shown by PCR using specific primers (Table 1).

**TABLE 1:** Infection efficiency of GGVA and GGVA-D alone or combined infection in *N. benthamiana* plants.

Inoculum	Experiment I	Experiment II	Experiment III
GGVA	20/20^a^	30/30	40/40
GGVA + GGVA-D	20/20	30/30	40/40
GGVA-D	0/20	0/30	0/40

*^a^Numbers of plants systemically infected/number of plants inoculated. Infection was determined using PCR.*

### Analysis of the Effect of GGVA-D on the Accumulated Level of GGVA

The effect of GGVA-D on the accumulation of GGVA was determined in the uninoculated apical leaves using qPCR. Twenty plants infected by GGVA alone or co-infected by GGVA/GGVA-D were randomly selected at 30 dpi. The results showed that the accumulated level of GGVA was significantly higher in GGVA/GGVA-D co-infected plants compared with that in GGVA infected plants (Figure 2D). Southern blotting hybridization validated the results of qPCR, indicating that GGVA-D enhances the accumulation level of GGVA DNA (Figure 2E).

### Screening for Potential Virulence Factors Encoded by GGVA

**FIGURE 3:**
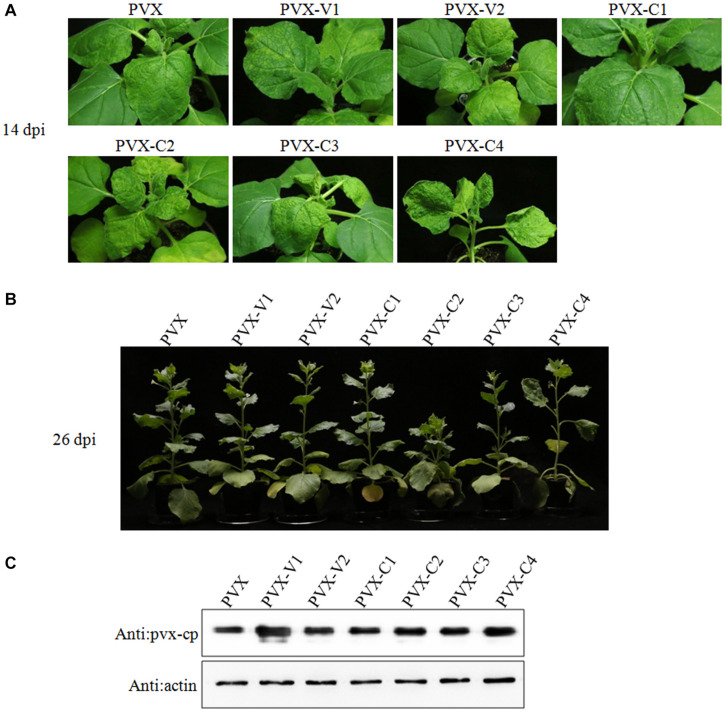
Transient expression of individual GGVA genes from potato virus X (PVX)-based vectors after infiltration of *N. benthamiana* plants. **(A,B)** Symptoms produced in *N. benthamiana* plants agroinfiltrated with PVX-based vector harboring GGVA-encoded proteins (V1, V2, C1, C2, C3, and C4), respectively. Plants were photographed at 14 dpi or 26 dpi. **(C)** The CP protein of PVX detected by western blotting using a polyclonal antibody against PVX CP. Actin, which is stably expressed in plants, was used as the internal reference.

To determine if any protein encoded by GGVA functions as a pathogenicity determinant, six ORFs were transiently expressed in *N. benthamiana* via a PVX-based heterologous expression system. By 14 dpi, the *N. benthamiana* plants inoculated with PVX-C2 developed upper leaf mottling and clustering symptoms and the plants inoculated with PVX-C4 developed stem elongating, upper leaf yellowing, and curling symptoms (Figure 3A). Overexpression of V1, V2, C1, and C3 proteins from a PVX vector induced plants to develop symptoms similar to those associated with PVX infection (Figure 3A). By 26 dpi, the *N. benthamiana* plants inoculated with PVX-C2 showed dwarfism (Figure 3B). To confirm the stability of the PVX-derived constructs, the expression of the coat protein (CP) in the uninoculated apical leaves was detected using western blotting with antibodies against PVX CP in each inoculation combination (Figure 3C). The results confirmed PVX infection via the recombinant vectors. In addition, the expression of individual ORFs was validated by RT-PCR using specific primers targeting individual encoding genes (data not shown).

### Identification of an RNA Silencing Suppressor Encoded by GGVA

**FIGURE 4:**
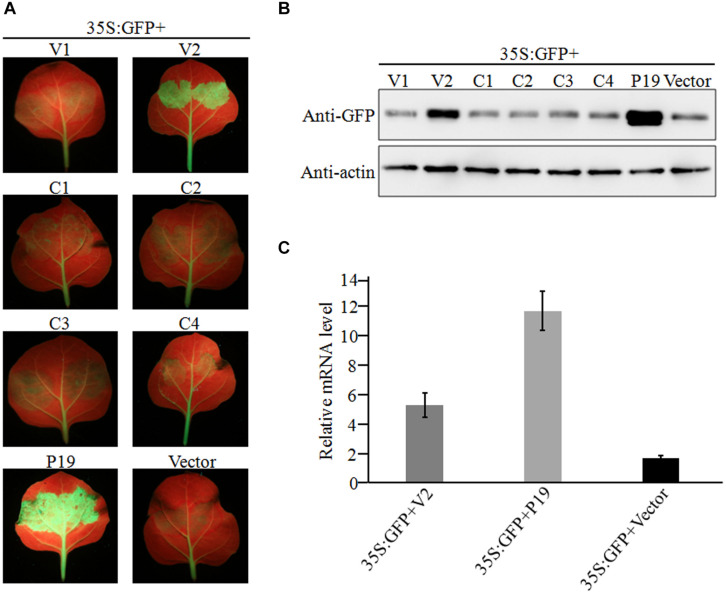
Suppression of local *GFP* silencing in 16C transgenic plant by GGVA V2 protein. **(A)** 35S:V1, 35S:V2, 35S:C1, 35S:C2, 35S:C3, 35S:C4, 35S:P19 (positive control) and empty vector pCHF3 (negative control) were co-infiltrated into *N. benthamiana* 16C plants with 35S-GFP, respectively. Fluorescence was observed and photographed under UV lamp at 7 dpi. **(B)** Western blotting analysis of GFP protein extracted from agroinfiltrated patches of 16C leaves shown in **(A)** using a GFP monoclonal antibody. Actin was used as internal reference. **(C)** A qRT-PCR assay was used to analyze the transcription level of GFP extracted from agroinfiltrated patches of 16C leaves shown in **(A)**. GADHP was used as the internal reference gene.

Geminiviruses can encode one or more proteins that function as suppressors of post-transcriptional gene silencing (PTGS). To investigate whether any proteins encoded by GGVA can suppress PTGS, six ORFs were introduced into binary vector pCHF3, separately. Leaves of 16C transgenic *N. benthamiana* plants carrying *GFP* (a gift from Professor David Baulcombe, University of Cambridge, United Kingdom) were agroinfiltrated with a mixture of a plasmid carrying a full length GFP insert under the control of the cauliflower mosaic virus 35 promoter (35S-GFP; Xiong et al., 2009) and either a test or a control pCHF3-derived construct. By 7 dpi, strong green fluorescence was only seen in the leaves co-infiltrated with 35S-GFP plus 35S-V2 or 35S-P19 carrying tomato bushy stunt virus p19 (Xiong et al., 2009; Figure 4A). As shown by western blotting (Figure 4B), the expression level of GFP in leaves co-infiltrated with 35S-GFP plus 35S-V2 was significantly higher than that in the negative control 35S-GFP plus empty vector. However, its expression was relatively weaker than that of the positive control co-infiltrated with 35S-GFP plus 35S-P19 (Figure 4B). *GFP* mRNA accumulated to higher levels in leaves co-infiltrated with 35S-GFP plus 35S-V2 compared with that in the negative control 35S-GFP plus empty vector, as shown by qRT-PCR, although it was much lower than that in leaves co-infiltrated with 35S-GFP plus 35S-P19 (Figure 4C). Taken together, these results suggest that protein V2 encoded by GGVA is a PTGS suppressor.

### Detection of Different Isolates of GGVA and Its Associated GGVA-D Defective DNA in Grapevine Samples

From June 2018 to June 2019, 18 grapevine samples (13 showing virus-like symptoms and 5 showing no symptoms) in vineyards from Pudong, Shanghai and Yuanmou, Yunnan were assessed for the presence of GGVA/GGVA-D through RCA combined with *Bam*HI digestion. The resulting DNA fragments were cloned and sequenced to confirm the virus identity. A BLASTn search confirmed that 11 samples were infected by GGVA (GenBank accession numbers: MT344704-344714), and 9 of the 11 samples were associated with the GGVA-D component (GenBank accession numbers: MT344716-344724), indicating that GGVA is prevalent in China and is frequently associated with the GGVA-D component. It is worth noting that the genome size of most GGVA isolates was stable at 2905 bp. However, the complete full-length sequence of their associated GGVA-D components was variable, ranging from 1549 to 1569 bp.

**FIGURE 5:**
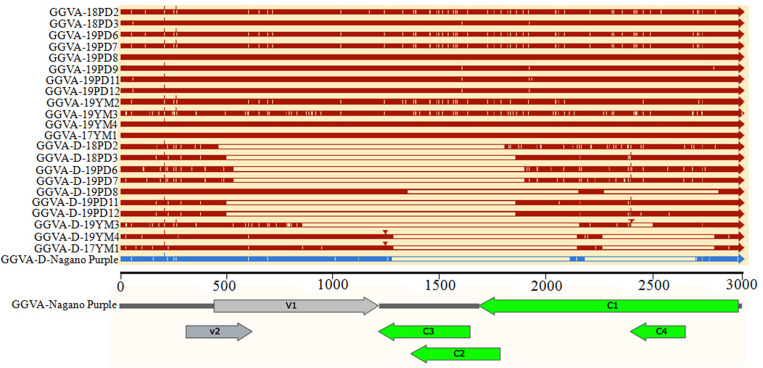
Comparison of the genome structure of different GGVA isolates and their associated GGVA-D components collected from Shanghai and Yunnan with that of GGVA isolate Nagano Purple from South Korea and its GGVA-D using software SnapGene.

The nucleotide sequence identity among 12 GGVA isolates from Shanghai and Yunnan ranged from 97.1 to 100%. The nucleotide sequence alignment with other GGVA isolates deposited in the NCBI database revealed an identity from 96.9 to 99.9% (Supplementary Figure S1). Interestingly, the 10 GGVA-D isolates obtained in our study shared a very high sequence identity with GGVA (>95%), with different GGVA-D isolates consisting of assemblies of different portions of the GGVA genome (Figure 5).

Further analysis of the genomic structure using the software SnapGene allowed us to conclude that all full-length nucleotide sequences from GGVA isolates encoded a total of six ORFs (Figure 5). However, compared with the sequence of GGVA-D-Nagano Purple (the sole reported sequence of GGVA-D in the NCBI database), the genomic structure of the GGVA-D components isolated in our study showed various deletions in the genome of the parental virus, although they are typically half the size of the full-length genome of GGVA (Figure 5).

### Population Variation Rate and Evolutionary Analysis of GGVA

**FIGURE 6:**
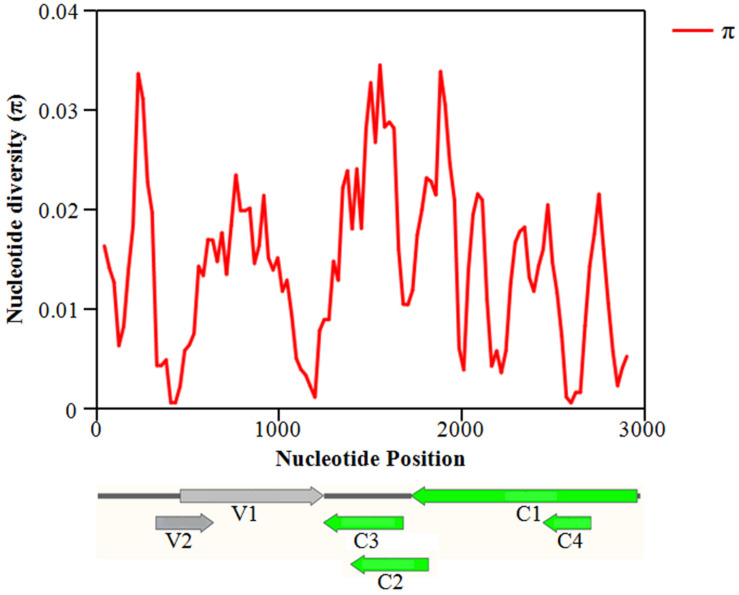
Distribution of molecular variation along the whole genome of GGVA population estimated by the nucleotide diversity (Pi). GGVA genome structure is used as the indicator showing the variation rate of each ORF region. The window length was set 100 nt wide with a 25 nt step size.

**FIGURE 7:**
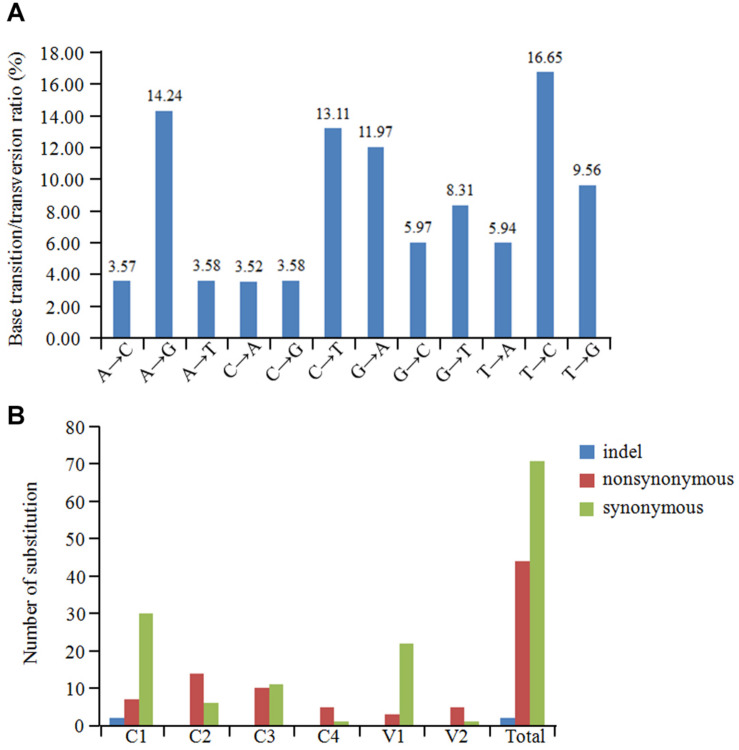
Population variation rate and evolutionary analysis of GGVA. **(A)** Maximum composite likelihood estimations of base transition/transversion ratios for the GGVA genome. The above ratios were calculated for each mutation type. **(B)** The number of substitution (indel, non-synonymous and synonymous) in six ORFs of GGVA genome was shown, respectively.

The overall nucleotide diversity (π) at all sites along the GGVA genome was evaluated using the DnaSP software. The variability rates for the full-length GGVA genome were below 4%. Meanwhile the average pairwise number of π was 0.01441, with the highest peak located in the C terminus of the C1 coding region and the lowest variation rate coexisted in the coding regions of C1 and C4 ORFs (Figure 6). Simultaneously, the maximum composite likelihood method was used to analyze the ratios of transitions and transversions at all sites along the GGVA genome. The results revealed that transitions of A↔G (26.21%) and C↔T (29.76%) occurred more frequently than transversions of A↔C (7.09%), A↔T (9.52%), C↔G (9.55%), and G↔T (17.87%) (Figure 7A), indicating a mutational bias for A↔G and C↔T transitions.

To estimate amino acid mutations in all GGVA isolates, the number of synonymous mutations, non-synonymous mutations, and indels in each GGVA ORF were estimated. Two indels were found in the C1 ORF, while no indels were observed in the other ORFs (C2, C3, C4, V1, and V2). A total of 7, 14, 10, 5, 3, and 5 non-synonymous mutations occurred in ORFs C1, C2, C3, C4, V1, and V2, respectively. Furthermore, the numbers of synonymous mutations in ORFs C1, C2, C3, C4, V1, and V2 were 30, 6, 11, 1, 22, and 1, respectively. These findings showed that synonymous mutations mainly occurred in the C1, C3, and V1 ORFs, while non-synonymous mutations mainly occurred in ORFs C2, C4, and V2. Additionally, the occurrence of indels in all six ORFs was very low (Figure 7B).

The type of selection acting on the six GGVA ORFs was investigated using the Datamonkey website. The dn/ds ratios were calculated based on the FEL, SLAC, and FUBAR methods. The results showed that the dn/ds ratios in ORFs V1, C1, C2, and C3 were significantly less than 1, suggesting that these four ORFs were under negative selection. The dn/ds ratio in V2 ORF was 2.24, which was significantly greater than 1, suggesting that V2 was under much stronger positive selection. Similarly, C4 ORF was also under positive selection (Table 2).

**TABLE 2:** Estimation of selection on the GGVA genes.

Gene	FEL		SLAC		FUBAR		dn/ds
	PS	NS	PS	NS	PS	NS	
V1	0	21	0	6	1	17	0.0808
V2	0	0	0	0	3	0	2.24
C1	1	39	0	11	4	26	0.182
C2	0	6	0	3	1	4	0.454
C3	1	6	0	3	3	4	0.477
C4	0	1	0	0	1	0	1.03

*PS, episodic positive/diversifying selected sites; NS, episodic negative/purifying selected sites. dn/ds, the average ratio between non-synonymous and synonymous substitutions for each pair of comparisons. A dn/ds < 1 indicates that the gene is under negative selection, whereas a dn/ds > 1 shows that the gene is under positive selection. These were used with a statistical significance level of P-value = 0.1 for FEL and SLAC and P-value = 0.9 for FUBAR. FEL (Fixed Effects Likelihood) uses a maximum-likelihood (ML) approach to infer non-synonymous (dn) and synonymous (ds) substitution rates on a per-site basis; SLAC (Single-Likelihood Ancestor Counting) uses a combination of maximum- likelihood (ML) and counting approaches to infer non-synonymous (dn) and synonymous (ds) substitution rates on a per-site basis; and FUBAR (Fast, Unconstrained Bayesian AppRoximation) uses a Bayesian approach to infer non-synonymous (dn) and synonymous (ds) substitution rates on a per-site basis.*

### Phylogenetic Analysis

**FIGURE 8:**
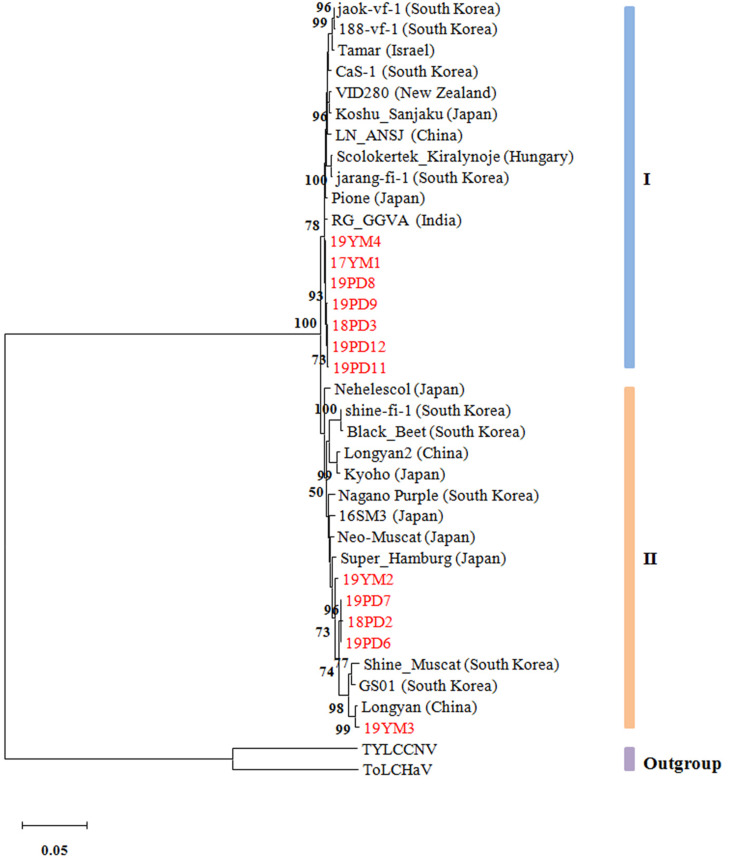
Phylogenetic tree based on the alignment of the GGVA complete genome nucleotide sequences. The tree was constructed using maximum-likelihood method in MEGA X and evaluated by a bootstrap test of 1000 replicates. The scare bar indicates the number of changes per site. Nodes with bootstrap values >50% are indicated. The complete sequences of tomato leaf curl Madagascar virus (ToLCMGV) and tomato yellow leaf curl China virus (TYLCCNV) served as outgroups.

To reveal the evolutionary relationships of different GGVA isolates, a phylogenetic tree was constructed based on the complete nucleotide sequences. The results showed that the 35 GGVA isolates could be divided into two evolutionary clades each with 18 or 17 isolates, respectively. Interestingly, GGVA isolates from the same geographical region were placed in different clades. For example, isolates 18PD2, 19PD6, and 19PD7 collected from Pudong were in group II and were more closely related to the GGVA isolates derived from Longyan in China and Korea (GS01 and Shine_Muscat). Isolates 18PD3, 19PD8, 19PD9, 19PD11, and 19PD12 clustered in group I together with isolates 17YM1 and 19YM4 from Yunnan, and were closer to isolates from India (RG_GGVA) and Japan (Pione). Similarly, GGVA Yunnan isolates like 19YM2 and 19YM3 belonged to group II, while isolate 17YM1 and 19YM4 belonged to group I. Thus, the GGVA isolates showed no obvious regional distribution characteristics (Figure 8).

## Discussion

Geminiviruses usually infect herbaceous plants, however, following the development of high throughput sequencing, more geminiviruses have been identified from woody plants. Citrus chlorotic dwarf-associated virus (CCDaV) (Loconsole et al., 2012), GRBV (Al Rwahnih et al., 2013), jatropha curcas mosaic virus (JaMV) (Wang et al., 2014), mulberry mosaic dwarf-associated virus (MMDaV) (Ma et al., 2015), and apple geminivirus (AGV) (Liang et al., 2015) have been reported. GGVA, which was recently discovered from two table grape cultivars from Korea, is a tentative species belonging to the family *Geminiviridae* (Al Rwahnih et al., 2017). In this study, we characterized several new GGVA isolates from grapevine samples collected from Shanghai and Yunnan Province, China. Previous reports indicated that GGVA was present in Liaoning, Shandong, Ningxia, Sichuan, Zhejiang, and Fujian provinces of China (Al Rwahnih et al., 2017; Fan et al., 2017). These results, combined with those of the present study, suggest that GGVA is widely distributed in China.

Previous evidence indicated that begomovirus populations exhibit a relatively high degree of within-species genetic variation and evolve rapidly similar to many RNA viruses, and that the variability across the DNA-A genome was not evenly distributed (Ge et al., 2007; Duffy and Holmes, 2009; Lima et al., 2017). The genetic variability estimated for tomato yellow leaf curl virus (TYLCV) based on its nucleotide diversity (π) was 0.04049, from the analysis of 222 full-length DNA-A sequences (Lima et al., 2017). Interestingly, the genetic variability of cotton leaf curl Gezira virus (CLCuGV) (*n* = 39, π = 0.04290) was statistically similar to that of TYLCV, although a small number of isolates was studied. Meanwhile, the genetic variability estimated for tomato yellow leaf curl China virus (TYLCCNV) was much higher (π = 0.10414) from analysis of 26 full-length DNA-A sequences (Lima et al., 2017). In the present study, an alignment of the complete genome sequences of 12 GGVA isolates obtained from Shanghai and Yunnan, and other 23 isolates downloaded from the NCBI database, showed that the nucleotide identity was over 97%, indicating a close evolutionary origin. Further analysis of the variation rate of single nucleotides across the entire GGVA genome identified low genetic variability, with π = 0.01441, suggesting that the GGVA population is less variable than most geminivirus populations, with ORFs V2 and C4 as the most conserved regions in the GGVA genome. Noticeably, the genetic variability of begomovirus populations was reported to be associated with an evolutionary adaptation to various plant species. Previous studies confirmed that the weed-infecting macroptilium yellow spot virus (MaYSV) (π = 0.0658) was more variable compared with the crop-infecting tomato severe rugose virus (ToSRV) (π = 0.0084) (Gonzalez-Aguilera et al., 2012; Lima et al., 2013), while a higher degree of genetic variation in isolates from MaYSV populations (π = 0.0622) than bean golden mosaic virus (BGMV) populations (π = 0.0067) was observed in common beans (Sobrinho et al., 2014). Taken together with other cases such as euphorbia yellow mosaic virus (EuYMV) in *Euphorbia heterophylla* (π = 0.02) (Mar et al., 2017) and ToSRV in tomato (π = 0.0102) (Rocha et al., 2013), these findings suggest that viruses that are well adapted to their hosts have a low degree of genetic variability and viruses that seem to have spilled over recently and are still adapting to a new host have much higher levels of genetic variability. Thus, our results for GGVA suggest that this virus is well adapted to grapevine.

The high genetic variability of geminivirus populations is predominantly driven by their high mutational dynamics combined with recombination (Duffy and Holmes, 2008, 2009; Lima et al., 2017). The analysis of base substitutions showed that the GGVA genome has a mutational bias for A↔G and C↔T transitions. Our findings were consistent with previous studies, which indicated that the genome of TYLCV or east African cassava mosaic virus (EACMV) contained a bias for transitions over transversions (Duffy and Holmes, 2008; Yang et al., 2014). Moreover, the main variation type in GGVA ORFs was synonymous substitutions. Noticeably, the variation produced by mutation and recombination depends on genetic drift and selection. We discovered that negative selection was acting on four ORFs (V1, C1, C2, and C3), while V2 and C4 were under positive selection. Consistently, negative selection was dominant for the whole genome of EuYMV, although there were three sites in the genome that were under positive selection (Mar et al., 2017).

A phylogenetic tree showed that the GGVA populations could be divided into two groups. However, in contrast to most geminiviruses or some RNA viruses (Rocha et al., 2013; Yang et al., 2018), this observation revealed some GGVA isolates from the same geographical region were located in different clades. This might be explained by the fact that the vegetative propagation of grapevine favors viral accumulation in the propagation material, and subsequently this virus is disseminated globally with the grapevine material. This was also noted for geminiviruses in sweet potato (Albuquerque et al., 2012). However, we do not rule out that this conclusion is tentative given the limited number of GGVA sequences used for cluster formation. Therefore, more GGVA isolate sequences from different other geographical sources should be added to determine whether GGVA phylogeny is related to geographical origin.

Some monopartite geminiviruses are frequently associated with satellite molecules (DNAβ or DNAα) that are half the size of the helper virus. These satellite molecules show low nucleotide sequence identity with the genome of their helper viruses (Cui et al., 2004; Patil and Dasgupta, 2006). Interestingly, one of the GGVA isolates had been reported to be accompanied by a defective DNA component, named as GGVA-D, which derived from the helper virus and contained about half the size of the parental genome (Al Rwahnih et al., 2017). Previously, some defective DNA molecules resulting from recombination between geminivirus and satellites had been identified (Huang et al., 2013). In the present study, we found GGVA-related defective DNA molecules in seven Shanghai and two Yunnan grapevine samples, suggesting that GGVA-D is present widely in China. Similarly, GGVA-D sequences contain a deletion of approximately 50% of the GGVA viral genome, and no non-viral sequences appear on these GGVA-D isolates.

In field grapevine samples, GGVA usually exists as a complex infection with multiple viruses (Blouin et al., 2018). Hence, it is difficult to identify the disease symptoms caused by GGVA alone. Previous evidence showed that a reverse genetics system based on infectious clones from the virus genome could advance our understanding of the pathogenicity of viruses and elucidate the host-virus-vector interaction (Ahlquist et al., 1984, 1987; Jarugula et al., 2018; Jiang et al., 2019). Here, we obtained a single virus source of GGVA through the construction of GGVA infectious clones. Our results confirmed that GGVA infected *N. benthamiana* plants and caused dwarfing and leaf edge curling symptoms. Similar results were reported for other grape-infecting viruses, such as grapevine leafroll-associated virus 3, which was successfully introduced into *N. benthamiana* via agroinfiltration (Jarugula et al., 2018). The key point of fulfilling Koch’s postulates is to identify causative relationship between a plant pathogen and plant disease in the natural host. However, experiments to demonstrate the infectivity of GGVA on the original host, grapevines, have been unsuccessful. Recent research revealed that infectious clones of GRBV could be agroinoculated into tissue cultured grapevine by vacuum-assisted infiltration and reproduced red blotch disease (Yepes et al., 2018). Furthermore, the authors tested whether different cultivars and rootstock genotypes could be infected by GRBV (Yepes et al., 2018). Hence, further experiments to gain important insights into GGVA infection on grapevines are required, possibly using the infectious clone of GGVA on a range of different cultivars.

Previous reports revealed that some satellites played important roles in regulating symptom development and virus accumulation (Cui et al., 2004; Patil and Dasgupta, 2006). In contrast to these previous findings, in the present study, we confirmed that GGVA-D co-infection together with GGVA did not aggravate the symptoms in *N. benthamiana* plants caused by GGVA single infection; however, to some extent, the presence of the GGVA-D led to an increase in the accumulation of GGVA. Defective DNA molecules or satellites associated with geminiviruses might have diverse roles in regulating the pathogenicity of helper viruses. Whether GGVA-D affects the spread of the helper virus in the field will be addressed in subsequent studies.

Serious diseases caused by geminivirus infection are often associated with viral-encoded symptom-determining factors, which usually function as suppressors of RNA silencing (Yang et al., 2011; Li et al., 2014). In this study, we screened for potential virulence factors and RNA silencing suppressors encoded by GGVA. The results showed that the C2 and C4 proteins have a decisive effect on the pathogenicity of PVX in *N. benthamiana* plants, and V2 has a strong PTGS suppressor activity. Several geminivirus C4 proteins induce severe developmental abnormalities in plants (Zeng et al., 2018; Fondong, 2019). A recent study by Mei et al. (2018) found that phosphorylation and N-myristoylation of tomato leaf curl Yunnan virus C4 was critical for viral pathogenicity. Li et al. (2018) found that S-acylation of C4 was essential for beet severe curly top virus symptom determination. The C2 protein of tomato leaf curl Taiwan virus was shown to be an important pathogenicity determinant, which interferes with host DNA methylation (Tu et al., 2017). To counteract RNA silencing defenses in plant, many geminivirus V2 proteins have been shown to serve as RNA silencing suppressors (Yang et al., 2018). Glick et al. (2008) confirmed that the interaction between suppressor of gene silencing 3 (SGS3) and V2 was required for the suppressor activity of V2 encoded by TYLCV. Further efforts are underway to elucidate the molecular mechanisms of GGVA infection by understanding the interaction between the host and V2, C2, and C4 proteins.

## Data Availability Statement

The datasets presented in this study can be found in online repositories. The names of the repository/repositories and accession number(s) can be found in the article/Supplementary Material.

## Author Contributions

SS, YH, and GJ performed the experiments. YQ and XZ participated in experimental design and coordination. YT, CY, and MD collected the field grapevines samples. SS drafted the manuscript. YQ proofread and finalized the manuscript. All authors have read and approved the final manuscript.

## Conflict of Interest

The authors declare that the research was conducted in the absence of any commercial or financial relationships that could be construed as a potential conflict of interest.

## Footnotes

1 www.datamonkey.org

2 http://unafold.rna.albany.edu/
