# Supplementary material for: Molecular Characterization and Genomic Function of Grapevine Geminivirus A
Source: Front Microbiol. 2020 Sep 2;11:555194. doi: 10.3389/fmicb.2020.555194 (PMC7493466; doi:10.3389/fmicb.2020.555194)
Supplement: Supplementary file 1 [file Presentation_1.pdf]

Supplementary Material

1. Supplementary Figures and Tables

1.1 Supplementary Figures

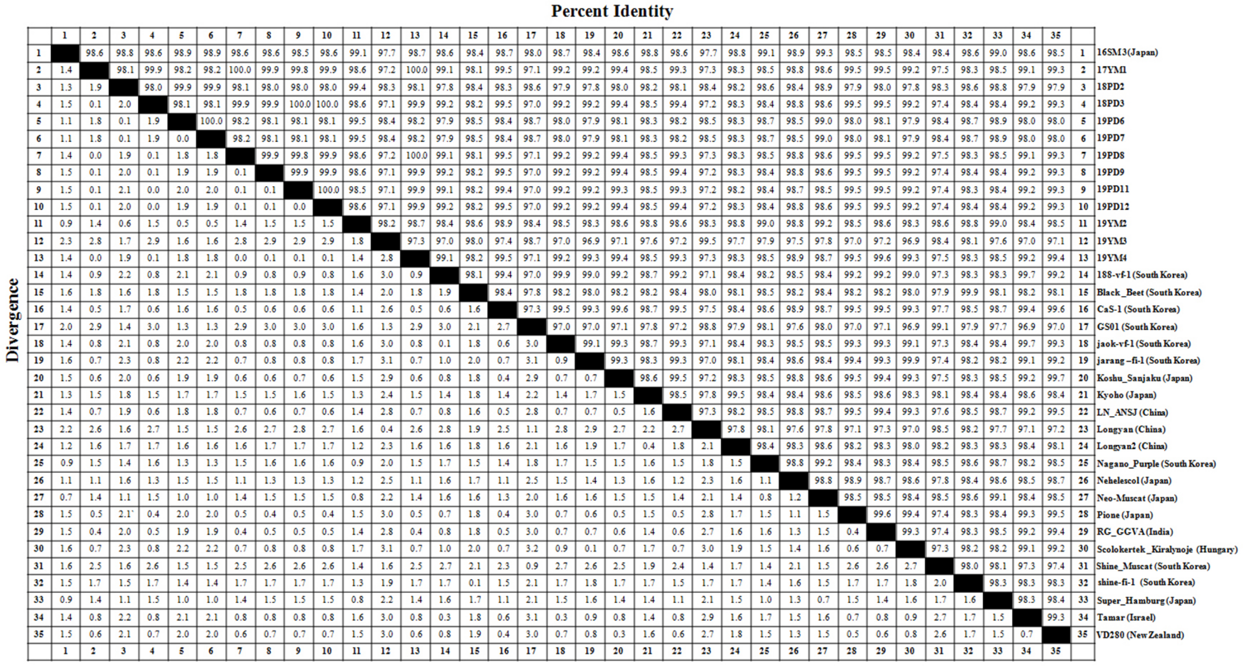

	1	2	3	4	5	6	7	8	9	10	11	12	13	14	15	16	17	18	19	20	21	22	23	24	25	26	27	28	29	30	31	32	33	34	35		
1		98.6	98.8	98.6	98.9	98.9	98.6	98.6	98.5	98.6	99.1	97.7	98.7	98.6	98.4	98.7	98.0	98.7	98.4	98.6	98.8	98.6	97.7	98.8	99.1	98.9	99.3	98.5	98.5	98.4	98.4	98.6	99.0	98.6	98.5	1	16SM3(Japan)
2	1.4		98.1	99.9	98.2	98.2	100.0	99.9	99.8	99.9	98.6	97.2	100.0	99.1	98.1	99.5	97.1	99.2	99.2	99.4	98.5	99.3	97.3	98.3	98.5	98.8	98.6	99.5	99.5	99.2	97.5	98.3	98.5	99.1	99.3	2	17YM1
3	1.3	1.9		98.0	99.9	99.9	98.1	98.0	98.0	98.0	99.4	98.3	98.1	97.8	98.4	98.3	98.6	97.9	97.8	98.0	98.2	98.1	98.4	98.2	98.6	98.4	98.9	97.9	98.0	97.8	98.3	98.6	98.8	97.9	97.9	3	18PD2
4	1.5	0.1	2.0		98.1	98.1	99.9	99.9	100.0	100.0	98.6	97.1	99.9	99.2	98.2	99.5	97.0	99.2	99.2	99.4	98.5	99.4	97.2	98.3	98.4	98.8	98.6	99.5	99.5	99.2	97.4	98.4	98.4	99.2	99.3	4	18PD3
5	1.1	1.8	0.1	1.9		100.0	98.2	98.1	98.1	98.1	99.5	98.4	98.2	97.9	98.5	98.4	98.7	98.0	97.9	98.1	98.3	98.2	98.5	98.3	98.7	98.5	99.0	98.0	98.1	97.9	98.4	98.7	98.9	98.0	98.0	5	19PD6
6	1.1	1.8	0.1	1.9	0.0		98.2	98.1	98.1	98.1	99.5	98.4	98.2	97.9	98.5	98.4	98.7	98.0	97.9	98.1	98.3	98.2	98.5	98.3	98.7	98.5	99.0	98.0	98.1	97.9	98.4	98.7	98.9	98.0	98.0	6	19PD7
7	1.4	0.0	1.9	0.1	1.8	1.8		99.9	99.8	99.9	98.6	97.2	100.0	99.1	98.1	99.5	97.1	99.2	99.2	99.4	98.5	99.3	97.3	98.3	98.5	98.8	98.6	99.5	99.5	99.2	97.5	98.3	98.5	99.1	99.3	7	19PD8
8	1.5	0.1	2.0	0.1	1.9	1.9	0.1		99.9	99.9	98.6	97.1	99.9	99.2	98.2	99.5	97.0	99.2	99.2	99.4	98.5	99.4	97.2	98.3	98.4	98.8	98.6	99.5	99.5	99.2	97.4	98.4	98.4	99.2	99.3	8	19PD9
9	1.5	0.1	2.1	0.0	2.0	2.0	0.1	0.1		100.0	98.5	97.1	99.9	99.1	98.2	99.4	97.0	99.2	99.2	99.3	98.5	99.3	97.2	98.2	98.4	98.7	98.5	99.5	99.5	99.2	97.4	98.3	98.4	99.2	99.3	9	19PD11
10	1.5	0.1	2.0	0.0	1.9	1.9	0.1	0.1	0.0		98.6	97.1	99.9	99.2	98.2	99.5	97.0	99.2	99.2	99.4	98.5	99.4	97.2	98.3	98.4	98.8	98.6	99.5	99.5	99.2	97.4	98.4	98.4	99.2	99.3	10	19PD12
11	0.9	1.4	0.6	1.5	0.5	0.5	1.4	1.5	1.5	1.5		98.2	98.7	98.4	98.6	98.9	98.4	98.5	98.3	98.6	98.8	98.6	98.3	98.8	99.0	98.8	99.2	98.5	98.6	98.3	98.6	98.8	99.0	98.4	98.5	11	19YM2
12	2.3	2.8	1.7	2.9	1.6	1.6	2.8	2.9	2.9	2.9	1.8		97.3	97.0	98.0	97.4	98.7	97.0	96.9	97.1	97.6	97.2	99.5	97.7	97.9	97.5	97.8	97.0	97.2	96.9	98.4	98.1	97.6	97.0	97.1	12	19YM3
13	1.4	0.0	1.9	0.1	1.8	1.8	0.0	0.1	0.1	0.1	1.4	2.8		99.1	98.2	99.5	97.1	99.2	99.3	99.4	98.5	99.3	97.3	98.3	98.5	98.9	98.7	99.5	99.6	99.3	97.5	98.3	98.5	99.2	99.4	13	19YM4
14	1.4	0.9	2.2	0.8	2.1	2.1	0.9	0.8	0.9	0.8	1.6	3.0	0.9		98.1	99.4	97.0	99.9	99.0	99.2	98.7	99.2	97.1	98.4	98.2	98.5	98.4	99.2	99.2	99.0	97.3	98.3	98.3	99.7	99.2	14	188-vf-1 (South Korea)
15	1.6	1.8	1.6	1.8	1.5	1.5	1.8	1.8	1.8	1.8	1.4	2.0	1.8	1.9		98.4	97.8	98.2	98.0	98.2	98.2	98.4	98.0	98.1	98.5	98.2	98.4	98.2	98.2	98.0	97.9	99.9	98.1	98.2	98.1	15	Black_Beet (South Korea)
16	1.4	0.5	1.7	0.6	1.6	1.6	0.5	0.6	0.6	0.6	1.1	2.6	0.5	0.6	1.6		97.3	99.5	99.3	99.6	98.7	99.5	97.5	98.4	98.6	98.9	98.7	99.5	99.5	99.3	97.7	98.5	98.7	99.4	99.6	16	CaS-1 (South Korea)
17	2.0	2.9	1.4	3.0	1.3	1.3	2.9	3.0	3.0	3.0	1.6	1.3	2.9	3.0	2.1	2.7		97.0	97.0	97.1	97.8	97.2	98.8	97.9	98.1	97.6	98.0	97.0	97.1	96.9	99.1	97.9	97.7	96.9	97.0	17	GS01 (South Korea)
18	1.4	0.8	2.1	0.8	2.0	2.0	0.8	0.8	0.8	0.8	1.6	3.0	0.8	0.1	1.8	0.6	3.0		99.1	99.3	98.7	99.3	97.1	98.4	98.3	98.5	98.5	99.3	99.3	99.1	97.3	98.4	98.4	99.7	99.3	18	jaok-vf-1 (South Korea)
19	1.6	0.7	2.3	0.8	2.2	2.2	0.7	0.8	0.8	0.8	1.7	3.1	0.7	1.0	2.0	0.7	3.1	0.9		99.3	98.3	99.3	97.0	98.1	98.4	98.6	98.4	99.4	99.3	99.9	97.4	98.2	98.2	99.1	99.2	19	jarang –fi-1 (South Korea)
20	1.5	0.6	2.0	0.6	1.9	1.9	0.6	0.6	0.7	0.6	1.5	2.9	0.6	0.8	1.8	0.4	2.9	0.7	0.7		98.6	99.5	97.2	98.3	98.5	98.8	98.6	99.5	99.4	99.3	97.5	98.3	98.5	99.2	99.7	20	Koshu_Sanjaku (Japan)
21	1.3	1.5	1.8	1.5	1.7	1.7	1.5	1.5	1.6	1.5	1.3	2.4	1.5	1.4	1.8	1.4	2.2	1.4	1.7	1.5		98.5	97.8	99.5	98.4	98.4	98.6	98.5	98.6	98.3	98.1	98.4	98.4	98.6	98.4	21	Kyoho (Japan)
22	1.4	0.7	1.9	0.6	1.8	1.8	0.7	0.6	0.7	0.6	1.4	2.8	0.7	0.8	1.6	0.5	2.8	0.7	0.7	0.5	1.6		97.3	98.2	98.5	98.8	98.7	99.5	99.4	99.3	97.6	98.5	98.7	99.2	99.5	22	LN_ANSJ (China)
23	2.2	2.6	1.6	2.7	1.5	1.5	2.6	2.7	2.8	2.7	1.6	0.4	2.6	2.8	1.9	2.5	1.1	2.8	2.9	2.7	2.2	2.7		97.8	98.1	97.6	97.8	97.1	97.3	97.0	98.5	98.2	97.7	97.1	97.2	23	Longyan (China)
24	1.2	1.6	1.7	1.7	1.6	1.6	1.6	1.7	1.7	1.7	1.2	2.3	1.6	1.6	1.8	1.6	2.1	1.6	1.9	1.7	0.4	1.8	2.1		98.4	98.3	98.6	98.2	98.3	98.0	98.2	98.3	98.3	98.4	98.1	24	Longyan2 (China)
25	0.9	1.5	1.4	1.6	1.3	1.3	1.5	1.6	1.6	1.6	0.9	2.0	1.5	1.7	1.5	1.4	1.8	1.7	1.5	1.5	1.6	1.5	1.8	1.5		98.8	99.2	98.4	98.3	98.4	98.5	98.6	98.7	98.2	98.5	25	Nagano_Purple (South Korea)
26	1.1	1.1	1.6	1.3	1.5	1.5	1.1	1.3	1.3	1.3	1.2	2.5	1.1	1.6	1.7	1.1	2.5	1.5	1.4	1.3	1.6	1.2	2.3	1.6	1.1		98.8	98.9	98.7	98.6	97.8	98.4	98.6	98.5	98.7	26	Nehelescol (Japan)
27	0.7	1.4	1.1	1.5	1.0	1.0	1.4	1.5	1.5	1.5	0.8	2.2	1.4	1.6	1.6	1.3	2.0	1.6	1.6	1.5	1.5	1.4	2.1	1.4	0.8	1.2		98.5	98.5	98.4	98.5	98.6	99.1	98.4	98.5	27	Neo-Muscat (Japan)
28	1.5	0.5	2.1`	0.4	2.0	2.0	0.5	0.4	0.5	0.4	1.5	3.0	0.5	0.7	1.8	0.4	3.0	0.7	0.6	0.5	1.5	0.5	2.8	1.7	1.5	1.1	1.5		99.6	99.4	97.4	98.3	98.4	99.3	99.5	28	Pione (Japan)
29	1.5	0.4	2.0	0.5	1.9	1.9	0.4	0.5	0.5	0.5	1.4	2.8	0.4	0.8	1.8	0.5	3.0	0.7	0.7	0.6	1.4	0.6	2.7	1.6	1.6	1.3	1.5	0.4		99.3	97.4	98.3	98.5	99.2	99.4	29	RG_GGVA (India)
30	1.6	0.7	2.3	0.8	2.2	2.2	0.7	0.8	0.8	0.8	1.7	3.1	0.7	1.0	2.0	0.7	3.2	0.9	0.1	0.7	1.7	0.7	3.0	1.9	1.5	1.4	1.6	0.6	0.7		97.3	98.2	98.2	99.1	99.2	30	Scolokertek_Kiralynoje (Hungary)
31	1.6	2.5	1.6	2.6	1.5	1.5	2.5	2.6	2.6	2.6	1.4	1.6	2.5	2.7	2.1	2.3	0.9	2.7	2.6	2.5	1.9	2.4	1.4	1.7	1.4	2.1	1.5	2.6	2.6	2.7		98.0	98.1	97.3	97.4	31	Shine_Muscat (South Korea)
32	1.5	1.7	1.5	1.7	1.4	1.4	1.7	1.7	1.7	1.7	1.3	1.9	1.7	1.7	0.1	1.5	2.1	1.7	1.8	1.7	1.7	1.5	1.7	1.7	1.4	1.6	1.5	1.7	1.7	1.8	2.0		98.3	98.3	98.3	32	shine-fi-1 (South Korea)
33	0.9	1.4	1.1	1.5	1.0	1.0	1.4	1.5	1.5	1.5	0.8	2.2	1.4	1.6	1.7	1.1	2.1	1.5	1.6	1.4	1.4	1.1	2.1	1.5	1.0	1.3	0.7	1.5	1.4	1.6	1.7	1.6		98.3	98.4	33	Super_Hamburg (Japan)
34	1.4	0.8	2.2	0.8	2.1	2.1	0.8	0.8	0.8	0.8	1.6	3.0	0.8	0.3	1.8	0.6	3.1	0.3	0.9	0.8	1.4	0.8	2.9	1.6	1.7	1.5	1.6	0.7	0.8	0.9	2.7	1.7	1.5		99.3	34	Tamar (Israel)
35	1.5	0.6	2.1	0.7	2.0	2.0	0.6	0.7	0.7	0.7	1.5	3.0	0.6	0.8	1.9	0.4	3.0	0.7	0.8	0.3	1.6	0.6	2.7	1.8	1.5	1.3	1.5	0.5	0.6	0.8	2.6	1.7	1.5	0.7		35	VD280 (New Zealand)
	1	2	3	4	5	6	7	8	9	10	11	12	13	14	15	16	17	18	19	20	21	22	23	24	25	26	27	28	29	30	31	32	33	34	35		

Supplementary Figure 1. The identity/divergence in nucleotide sequences among 35 different GGVA isolates using MegAlign software.

1.2 Supplementary Tables

Table S1 Nine samples collected from different fruit trees located in Yunnan regions of China

Number	Sampling location	Sampling name	Host
1	Luliang	LL3	Peach
2	Shilin	SL2	Apricot
3	Shilin	SL3.1	Peach
4	Shilin	SL3.2	Peach
5	Shilin	SL3.3	Peach
6	Shilin	SL3.4	Peach
7	Shilin	SL5	Citrus
8	Shilin	SL6	Walnut
9	Yuanmou	17YM1	Grapevine

Table S2 Information of grapevine samples collected from different regions of China

Number	Sampling name	Sampling location	Sampling year	Grape cultivar
1	18PD2	Pudong, Shanghai	2018 summer	/
2	18PD3	Pudong, Shanghai	2018 summer	/
3	19PD2	Pudong, Shanghai	2019 spring	Kyoho
4	19PD3	Pudong, Shanghai	2019 spring	Kyoho
5	19PD4	Pudong, Shanghai	2019 spring	Kyoho
6	19PD5	Pudong, Shanghai	2019 spring	Kyoho
7	19PD6	Pudong, Shanghai	2019 spring	Drunken Golden Grape
8	19PD7	Pudong, Shanghai	2019 spring	Drunken Golden Grape
9	19PD8	Pudong, Shanghai	2019 spring	Drunken Golden Grape
10	19PD9	Pudong, Shanghai	2019 spring	Chenxiang
11	19PD10	Pudong, Shanghai	2019 spring	Chenxiang
12	19PD11	Pudong, Shanghai	2019 spring	Chenxiang
13	19PD12	Pudong, Shanghai	2019 spring	Chenxiang
14	19YM1	Yuanmou, Yunnan	2019 summer	Chixia
15	19YM2	Yuanmou, Yunnan	2019 summer	Shine-Muscat
16	19YM3	Yuanmou, Yunnan	2019 summer	Red Grape
17	19YM4	Yuanmou, Yunnan	2019 summer	Shine-Muscat
18	19YM5	Yuanmou, Yunnan	2019 summer	Shine-Muscat

Table S3 Primers used in this study

Primer	Nucleotide sequence (5'–3')
Primers used for virus detection	
GGVA-1-F	ACCGGATGGCCGCCGATTTTT
GGVA-1-R	AATATTATAGGATGGCCGCTACTA
GGVA-1678-F	TCTTCTAGCTGGACTGTCTGTCTTG
GGVA-1678-R	AGAAGAAGGATCAATTGCCAGTGC
GGVA-440-F	GATCCATGGACTTCAATCCGAGAAAG
GGVA-440-R	CAATCTATGAGTTGTTGGGCCGTTC
Primers used for the construction of infectious clones	
GGVA-1-(2885)-F	GTCGACAGCGGCCATCCTATAATATTACCG
GGVA-1-(2884)-R	ACTAGTTCTGGGAGAGGTATATATTTC
GGVA-2-(2885)-F	GTCGACAGAACTAGTAGCGGCCATCCTATAATATTAC
GGVA-2-(2480)-R	GGTACCAGATGTCATCTCCTGGGGAG
GGVA-D1-(446)-F	GTCGACATGGACTTCAATCCGAGAAAGAGG
GGVA-D1-(445)-R	GGATCCAATCTATGAGTTGTTGGGC
GGVA-D2-(446)-F	GTCGACATGGGATCCATGGACTTCAATCCGAGAAAGAGG
GGVA-D2-(388)-R	GGTACCGAGGCAATATTTCAGCAGGATGAAGC
Primers used for the construction of expression vectors	
PVX -V1-AscI-F	GGCGCGCCATGGACTTCAATCCGAGAAAGAGGAAA
PVX -V1-SalI-R	GTCGACTTAATGATAATATATTATTCTACAATTCCAATCTCCTT
PVX -V2-AscI-F	GGCGCGCCATGTGGTTGGTGTTTATGGATCGC
PVX -V2-SalI-R	GTCGACTCAGGATATACTGACTGACTTGACCTCC
PVX -C1-AscI-F	GGCGCGCCATGGCCGCTACTAGTTCTGGGA
PVX -C1-SalI-R	GTCGACCTAGCTGGACTGTCTGTCTTGGC
PVX -C2-AscI-F	GGCGCGCCATGCAATTTTCGTCTCCCTGCAG
PVX -C2-SalI-R	GTCGACCTATTTTGTAAAGAGATCAGCCCAAATG
PVX -C3-AscI-F	GGCGCGCCATGCCTGTCGGCCATGTCAG
PVX -C3-SalI-R	GTCGACTTAATATAAATTGAATTTTACATCTGTTGAGTACATTTC
PVX -C4-AscI-F	GGCGCGCCATGGGCAACCGCATCTGCAT
PVX -C4-SalI-R	GTCGACTTAACGCCTCAGCATAAGCGTCG
enfusion-V1-pCHF3-KpnI-F	CGGGGGACGAGCTCGGTACCATGGACTTCAATCCGAGAAAGAG
enfusion-V1-pCHF3-BamHI-R	GTCGACTCTAGAGGATCCTTAATGATAATATATTATTCTACAATTCCAATC
enfusion-V2-pCHF3-KpnI-F	CGGGGGACGAGCTCGGTACCATGTGGTTGGTGTTTATGGATC
enfusion-V2-pCHF3-BamHI-R	GTCGACTCTAGAGGATCCTCAGGATATACTGACTGACTTGACC
enfusion-C1-pCHF3-KpnI-F	CGGGGGACGAGCTCGGTACCATGGCCGCTACTAGTTCTG
enfusion-C1-pCHF3-BamHI-R	GTCGACTCTAGAGGATCCCTAGCTGGACTGTCTGTCTTG
enfusion-C2-pCHF3-KpnI-F	CGGGGGACGAGCTCGGTACCATGCAATTTTCGTCTCCCTG
enfusion-C2-pCHF3-BamHI-R	GTCGACTCTAGAGGATCCCTATTTTGTAAAGAGATCAGCCCAAATG
enfusion-C3-pCHF3-KpnI-F	CGGGGGACGAGCTCGGTACCATGCCTGTCGGCCATG
enfusion-C3-pCHF3-BamHI-R	GTCGACTCTAGAGGATCCTTAATATAAATTGAATTTTACATCTGTTGAGTAC

enfusion-C4-pCHF3-KpnI-F	CGGGGGACGAGCTCGGTACCATGGGCAACCGCATCTG
enfusion-C4-pCHF3-BamHI-R	GTCGACTCTAGAGGATCCTTAACGCCTCAGCATAAGCG
Primers used for qPCR	
qPCR-GGVA-1560-F	GTATACTCCTTTCTGGGTTTGCTGC
qPCR-GGVA-1780-R	GCAATTTTCGTCTCCCTGCAGGA
q25S-F	ATAACCGCATCAGGTCTCCA
q25S-R	CCGAAGTTACGGATCCATTT
qPCR-GFP-F	CTGTTCCATGGCCAACACTTGTCAC
qPCR-GFP-R	GCCATGATGTATACGTTGTGGGAGT
qPCR-GADPH-F	GCAGTGAACGACCCATTTATCTC
qPCR-GADPH-R	AACCTTCTTGGCACCACCCT

Table S4 Sequences of different GGVA isolates

Isolate name	GenBank accession number	Country	Genome size (nt)
17YM1	MT344703	China	2904
Neo-Muscat	KX570613.1	Japan	2905
Super Hamburg	KX570610.1	Japan	2906
16SM3	LC424098.1	Japan	2905
Nagano Purple	KX570607.1	South Korea	2905
shine-fi-1	MF163265.1	South Korea	2905
Nehelescol	KX570615.1	Japan	2905
Black Beet	KX570609.1	South Korea	2906
CaS-1	MF163262.1	South Korea	2905
Longyan	KX570611.1	China	2904
Shine Muscat	KX570614.1	South Korea	2903
Kyoho	KX570612.1	Japan	2905
Longyan2	KX574323.1	China	2903
LN_ANSJ	KX950822.1	China	2905
Koshu Sanjaku	KX570617.1	Japan	2905
Pione	KX570616.1	Japan	2905
jaok-vf-1	MF163263.1	South Korea	2905
Tamar	KX618694.1	Israel	2905
188-vf-1	MF163261.1	South Korea	2905
jarang-fi-1	MF163264.1	South Korea	2905
Scolokertek-Kiralynoje	KX570618.1	Hungary	2905
GS01	MK336184.1	South Korea	2904
VID280	MK690474.1	New Zealand	2905
RG_GGVA	MN661401.1	India	2905
